# The relationship between relational continuity and family physician follow-up after an antidepressant prescription in older adults: a retrospective cohort study

**DOI:** 10.1186/s12875-024-02361-0

**Published:** 2024-04-22

**Authors:** David Rudoler, Natasha Lane, Agnes Grudniewicz, Vicki Ling, David Snadden, Therese A Stukel

**Affiliations:** 1 Faculty of Health Sciences, Ontario Tech University, Oshawa, Ontario Canada; 2 Ontario Shores Centre for Mental Health Sciences, Whitby, Ontario Canada; 3 ICES Central, Toronto, Ontario Canada; 4 Division of Geriatric Medicine, University of Toronto, Toronto, Ontario Canada; 5 Telfer School of Management, University of Ottawa, Ottawa, Ontario Canada; 6 University of British Columbia Northern Medical Program, Prince George, British Columbia, Canada; 7 Institute of Health Policy, Management and Evaluation, University of Toronto, Toronto, Ontario Canada

**Keywords:** Primary care, Antidepressant, Follow-up care, Continuity of care, Family medicine, Administrative health data

## Abstract

**Background:**

Side effects can occur within hours to days of starting antidepressant medications, whereas full therapeutic benefit for mood typically takes up to four weeks. This mismatch between time to harm and lag to benefit often leads to premature discontinuation of antidepressants, a phenomenon that can be partially reversed through early doctor-patient communication and follow-up. We investigated the relationship between relational continuity of care – the number of years family physicians have cared for older adult patients – and early follow-up care for patients prescribed antidepressants.

**Methods:**

A retrospective cohort study was conducted on residents of Ontario, Canada aged 66 years or older who were dispensed their first antidepressant prescription through the provincial drug insurance program between April 1, 2016, and March 31, 2019. The study utilized multivariable regression to estimate the relationship between relational continuity and 30-day follow-up with the prescribing family physician. Separate estimates were generated for older adults living in urban, non-major urban, and rural communities.

**Results:**

The study found a small positive relationship between relational continuity of care and follow-up care by the prescribing family physician for patients dispensed a first antidepressant prescription (RRR = 1.005; 95% CI = 1.004, 1.006). The relationship was moderated by the patients’ location of dwelling, where the effect was stronger for older adults residing in non-major urban (RRR = 1.009; 95% CI = 1.007, 1.012) and rural communities (RRR = 1.006; 95% CI = 1.002, 1.011).

**Conclusions:**

Our findings do not provide strong evidence of a relationship between relational continuity of care and higher quality management of antidepressant prescriptions. However, the relationship is slightly more pronounced in rural communities where access to continuous primary care and specialized mental health services is more limited. This may support the ongoing need for the recruitment and retention of primary care providers in rural communities.

**Supplementary Information:**

The online version contains supplementary material available at 10.1186/s12875-024-02361-0.

## Background

Each year, 4 to 17% of older adults begin taking antidepressants to alleviate symptoms related to depression, anxiety, pain syndromes, or sleep disturbances, with selective serotonin reuptake inhibitors (SSRIs) being the most commonly prescribed [1, 2]. For most indications, antidepressants should be taken for at least six months. A study by Lin et al. [3] suggests that nearly 30% of patients stop taking these medications during the first month of therapy, and over 40% stop taking them by the third month, with physician-patient communication being one of the drivers of patients’ resulting views on medication discontinuation.

Canadian guidelines for depression management state that patients should see a physician (preferably the prescriber) within two weeks of antidepressant initiation [4]. Follow-up by physicians after the initial prescription of an antidepressant has been linked to better patient outcomes, including higher patient satisfaction with care and reduced visits to the emergency department and hospitalizations [5–7]. Most patients receive a depression diagnosis and their first antidepressant prescription from a family physician (FP) [8], but studies have suggested that many patients often do not receive appropriate follow-up after the initial prescription. According to research using nationally representative survey data, only 55% of Canadians with depression reported receiving guideline-concordant depression care, and this rate was lower in rural settings and among patients with less complex illness [5].

One possible way to improve the delivery of appropriate care in primary care settings is to increase the continuity of care. Continuity of care has been associated with improvement in a variety of patient outcomes [9–12]. In particular, it is believed that relational continuity — an ongoing relationship between a provider and a patient where the provider gets to know the patient as a person [13–15] — leads to a better understanding of patient needs due to a trusting relationship between the provider and the patient [13]. However, there is little evidence regarding the relationship between relational continuity of care in primary care settings and medication management-related outcomes, specifically evidence-based depression care.

This study investigates the association between a measure of relational continuity between FPs and patients, and follow-up care for patients prescribed antidepressants, specifically, 30-day follow-up after a first antidepressant prescription. This is an important quality indicator in depression care and an essential element of effective interventions to increase antidepressant medication compliance [16], which is notoriously poor [17]. We hypothesize that longer periods of relational continuity will facilitate follow-up after antidepressant initiation. This hypothesis is based on evidence that relational continuity is associated with better follow-up care for other chronic conditions [18, 19]. We test this hypothesis separately for people living in urban and rural communities, as rural communities face greater challenges recruiting and retaining physicians over the longer term [20].

## Methods

This study was conducted in Ontario, the most populous province in Canada, where healthcare funding and delivery falls under provincial jurisdiction. In Ontario, FPs operate private practices and receive public payments through various payment models, including fee-for-service, capitation, and salary. Most prescription drugs are covered by the Ontario Drug Benefit Program (ODBP) for Ontario residents 65 years of age and older.

We conducted a retrospective cohort study of Ontario residents who were dispensed a first antidepressant prescription through the ODBP between April 1, 2016 and March 31, 2019 (see Supplementary Materials for the Drug Identification Number (DIN) list of included antidepressant medications). This period was chosen to exclude the COVID-19 pandemic. We excluded people who were not eligible for provincial health insurance or who did not reside in Ontario in the three years prior to the dispensing date. A first prescription was defined as one where no antidepressant had been dispensed in the previous 90 days. We did not limit our sample to individuals with a diagnosis of depression as administrative data are insensitive to this diagnosis [21], and antidepressants have the same side effect profile and need for follow-up regardless of their indication. To focus on the population eligible for provincial drug insurance, we also excluded people less than 66 years of age on the dispensing date. We also excluded people residing in long-term care or complex continuing care facilities and those who did not receive their antidepressant prescription from an eligible physician. To focus on prescriptions from physicians with ongoing relationships with patients, eligible physicians were limited to family medicine specialists. These physicians were designated as comprehensive family physicians for at least one year within three years of the dispensing date [22].

### Data sources

For this study, we utilized linked administrative health databases, including the ODBP claims database for information on the dispensing of publicly insured prescriptions; the Registered Persons Database for information and vital statistics for all individuals eligible to receive public health insurance in Ontario; the Canadian Institute for Health Information (CIHI) National Ambulatory Care Reporting System and the Discharge Abstract Database for information on emergency department visits and hospital separations; the Ontario Mental Health Reporting System for information on inpatient psychiatric care; the Ontario Health Insurance Program claims database for physician medical claims; the Corporate Provider Database for practice and demographic information on practicing FPs; and the Ontario Marginalization Index for neighborhood-level socioeconomic information. All records from these data sources were linked using unique encoded identifiers and analyzed at ICES (formerly the Institute for Clinical Evaluative Sciences). ICES is an independent, non-profit research institute whose legal status under Ontario’s health information privacy laws allows it to collect and analyze health care and demographic data, without consent, for health system evaluation and improvement. The use of data in this project is authorized under Sect. 45 of Ontario’s Personal Health Information Project Act (PHIPA) and does not require review by a Research Ethics Board.

### Outcome and exposure

Our primary outcome was an ambulatory visit with the prescribing FP within 30 days of the dispensing date of the first antidepressant prescription. A secondary outcome was an ambulatory visit with any FP within 30 days of the dispensing date. We defined our main exposure, relational continuity, as the number of years since the prescribing FP first billed for care of the patient prior to the initial prescription (up to 20 years).

### Confounders

We collected patient sociodemographic data, including age, biological sex, neighborhood-level material deprivation, rurality (as determined by the Rurality Index of Ontario (RIO) score [23]), and migration status (defined as registration with the provincial insurer within the past 10 years). We also recorded healthcare utilization variables, including the number of distinct medications dispensed in the previous 12 months, number of primary care visits in the prior 24 months, number of visits with the prescribing FP in the previous 10 years, and the percentage of primary care visits involving the prescribing FP over the past 10 years. To assess patient morbidity, we used the Canadian Institute for Health Information (CIHI) grouper to categorize patients into levels of need (major-palliative, moderate, minor, non-user, and no health condition). Lastly, we captured information about the prescribing FP, including their biological sex, age, and payment model.

### Statistical analysis

We report descriptive statistics for patients and FPs by the patient’s location of dwelling, specifically, urban (RIO < 10), non-major urban (10 < = RIO < 40), and rural (RIO > = 40) census subdivisions and assessed balance using standardized mean differences.

In multivariable regression analysis, we employed a modified Poisson estimator [24] within the framework of generalized estimating equations with an exchangeable correlation structure and robust standard errors to assess the conditional relationship between years of relational continuity (from 0 to 20 years) and 30-day physician follow-up. Our analysis consisted of three stages of model estimation. Model 1 incorporated patient-level sociodemographic variables including the interaction of age and sex categories, neighborhood-level material deprivation, and migration status and patient morbidity (CIHI grouper). Model 2 added the number of primary care visits in the prior 24 months and number of unique prescriptions at the dispensing date, and Model 3 added prescribing-physician-level variables (physician sex, physician age, and physician payment model).

## Results

We found 1,113,105 instances of antidepressant medications being dispensed to older adults who received their first prescription between April 1, 2016, and March 31, 2019. We excluded 9,630 cases due to ineligibility for provincial health insurance, 193 cases because the individuals were non-residents of Ontario, 49,212 cases because the prescriptions were given to individuals under 66 years of age at the time of dispensing, and 22,256 cases because the individuals were residing in a long-term care facility. Additionally, we excluded 179,327 cases where the antidepressant prescription was provided by a physician who was not a comprehensive FP. We retained the earliest prescription for each individual, resulting in the removal of 603,125 cases. This left 248,362 unique cases of older adults receiving a first antidepressant prescription. In terms of breakdown by geography, 161,912 lived in urban areas, versus 63,001 in non-major urban areas, and 22,150 in rural areas.

In Table 1, we report baseline patient characteristics by patients’ location of dwelling. Compared to patients in urban communities, patients in rural communities were less likely to be 85 years and older (rural = 8.4%, urban = 11.7%), less likely to reside in the least materially deprived communities (rural = 9.9%, urban = 23.5%), less likely to be a recent migrant (rural = 1.2%, urban = 2.9%), less likely to see a psychiatrist in the previous 12 months (rural = 2.2%, urban = 4.3%), and had fewer primary care visits in the previous 24 months (mean +/- SD) (rural = 11.62 (± 8.69), urban = 14.96 (± 11.63)). However, these populations were similar (standardized mean differences < 0.1) for all other measured characteristics. With respect to the average years of relational continuity, differences between rural and urban communities were small (rural = 9.99 (± 7.45), urban = 10.44 (± 7.40)).

**Table 1:** Baseline patient characteristics by location of dwelling

Characteristic	Urban	non-major urban	Rural	Missing	Total	Standardized difference: non-major urban vs. Urban	Standardized difference: Rural vs. Urban	Standardized difference: Missing vs. Urban
	*N* = 161,912	*N* = 63,001	*N* = 22,150	*N* = 1,299	*N* = 248,362			
**Age group**								
66–69	57,566 (35.6%)	23,845 (37.8%)	8,675 (39.2%)	602 (46.3%)	90,688 (36.5%)	0.048	0.075	0.221
70–74	37,461 (23.1%)	15,392 (24.4%)	5,396 (24.4%)	324 (24.9%)	58,573 (23.6%)	0.03	0.029	0.042
75–79	27,688 (17.1%)	10,515 (16.7%)	3,768 (17.0%)	201 (15.5%)	42,172 (17.0%)	0.011	0.002	0.044
80–84	20,235 (12.5%)	7,054 (11.2%)	2,446 (11.0%)	106 (8.2%)	29,841 (12.0%)	0.04	0.045	0.143
85+	18,962 (11.7%)	6,195 (9.8%)	1,865 (8.4%)	66 (5.1%)	27,088 (10.9%)	0.061	0.11	0.241
**Sex**								
Female	110,728 (68.4%)	41,883 (66.5%)	14,332 (64.7%)	821 (63.2%)	167,764 (67.5%)	0.041	0.078	0.109
Male	51,184 (31.6%)	21,118 (33.5%)	7,818 (35.3%)	478 (36.8%)	80,598 (32.5%)	0.041	0.078	0.109
**Neighborhood material deprivation**								
1 - least deprived	38,108 (23.5%)	12,326 (19.6%)	2,195 (9.9%)	88 (6.8%)	52,717 (21.2%)	0.097	0.371	0.481
2	32,395 (20.0%)	14,808 (23.5%)	3,910 (17.7%)	149 (11.5%)	51,262 (20.6%)	0.085	0.06	0.236
3	29,863 (18.4%)	13,252 (21.0%)	5,947 (26.8%)	157 (12.1%)	49,219 (19.8%)	0.065	0.202	0.177
4	31,328 (19.3%)	11,562 (18.4%)	5,688 (25.7%)	285 (21.9%)	48,863 (19.7%)	0.025	0.152	0.064
5 - most deprived	29,766 (18.4%)	10,593 (16.8%)	4,053 (18.3%)	255 (19.6%)	44,667 (18.0%)	0.041	0.002	0.032
6 - missing	452 (0.3%)	460 (0.7%)	357 (1.6%)	365 (28.1%)	1,634 (0.7%)	0.064	0.138	0.869
**Recent migrant**	4,750 (2.9%)	670 (1.1%)	262 (1.2%)	45 (3.5%)	5,727 (2.3%)	0.134	0.124	0.03
**CIHI grouper**								
No health condition	1,435 (0.9%)	842 (1.3%)	390 (1.8%)	31 (2.4%)	2,698 (1.1%)	0.043	0.077	0.118
Major Palliative	46,983 (29.0%)	17,836 (28.3%)	6,120 (27.6%)	359 (27.6%)	71,298 (28.7%)	0.016	0.031	0.031
Moderate	80,463 (49.7%)	30,957 (49.1%)	10,719 (48.4%)	612 (47.1%)	122,751 (49.4%)	0.011	0.026	0.052
Minor	32,375 (20.0%)	13,114 (20.8%)	4,816 (21.7%)	280 (21.6%)	50,585 (20.4%)	0.02	0.043	0.038
Obstetrics and newborn*	--	--	--	--	--	--	--	--
Non-user	654 (0.4%)	251 (0.4%)	105 (0.5%)	17 (1.3%)	1,027 (0.4%)	0.001	0.011	0.098
**No. of unique medications at dispensing date**								
0–5	25,138 (15.5%)	10,439 (16.6%)	3,871 (17.5%)	211 (16.2%)	39,659 (16.0%)	0.028	0.053	0.02
6–9	39,811 (24.6%)	16,509 (26.2%)	5,858 (26.4%)	340 (26.2%)	62,518 (25.2%)	0.037	0.043	0.036
10–19	72,935 (45.0%)	27,929 (44.3%)	9,688 (43.7%)	563 (43.3%)	111,115 (44.7%)	0.014	0.026	0.034
20+	24,028 (14.8%)	8,124 (12.9%)	2,733 (12.3%)	185 (14.2%)	35,070 (14.1%)	0.056	0.073	0.017
**Saw psychiatrist in 12 months prior to dispensing date**	6,981 (4.3%)	1,462 (2.3%)	494 (2.2%)	23 (1.8%)	8,960 (3.6%)	0.111	0.117	0.148
**Primary care visits 24 month prior to dispensing date**								
Mean ± SD	14.96 ± 11.63	12.12 ± 9.03	11.62 ± 8.69	10.85 ± 7.64	13.92 ± 10.86	0.273	0.326	0.418
Median (IQR)	12 (8–19)	10 (6–16)	10 (6–15)	9 (5–14)	11 (7–18)			
**Visits to prescribing PCP in 10 years to dispensing date**								
Mean ± SD	7.61 ± 20.74	6.23 ± 20.29	5.96 ± 20.95	6.97 ± 28.29	7.11 ± 20.70	0.067	0.079	0.026
Median (IQR)	5 (3–8)	4 (3–6)	4 (2–6)	3 (2–5)	5 (3–7)			
**Percent of primary care visits with prescribing PCP in 10 years prior to dispensing date**								
Mean ± SD	59.24 ± 34.07	60.49 ± 34.79	57.89 ± 36.24	56.16 ± 36.98	59.42 ± 34.47	0.037	0.038	0.087
Median (IQR)	69 (26–91)	72 (27–93)	68 (21–93)	66 (18–92)	70 (26–91)			
**Relational continuity (years)**								
Mean ± SD	10.44 ± 7.40	10.33 ± 7.34	9.99 ± 7.45	9.45 ± 7.54	10.37 ± 7.39	0.014	0.060	0.132
Median (IQR)	10 (3–19)	10 (3–19)	9 (3–18)	9 (2–18)	10 (3–19)			

* This category was null given our study population included older adults ( > = 66 years)

In Table 2, we report baseline prescribing FP characteristics based on patients’ location of dwelling. These statistics are reported at the patient-level, which means that the same physician can be counted multiple times. Compared to patients in urban communities, patients in rural communities received their prescription from FPs with smaller patient panels (rural = 1,280 (± 678), urban = 1,597 (± 812)), within 15 years of graduation (rural = 25.8%, urban = 21.8%), or working in interdisciplinary practice models (rural = 53.4%, urban = 22.0%), and were less likely to receive a prescription from a full-time physician (rural = 68.1%, urban = 78.2%). Table 3 shows unadjusted rates of follow-up care. 34.08% of the cohort had a follow-up visit within 30 days with the prescribing FP. The rate of follow-up ranged from 35.92% in urban areas to 29.29% in rural areas. The rate of a visit with any physician within 30 days was (56.4%), with any FP (43.3%), and with a psychiatrist (1.1%) (Supplementary Table A1).

**Table 2:** Baseline prescribing family physician characteristics by patients’ location of dwelling

Characteristic	Urban	Non-major urban	Rural	Missing	Total	Standardized difference: non-major urban vs. Urban	Standardized difference: Rural vs. Urban	Standardized difference: Missing vs. Urban
	*N* = 161,912	*N* = 63,001	*N* = 22,150	*N* = 1,299	*N* = 248,362			
**Patient panel size**								
Mean ± SD	1,597.25 ± 812.19	1,578.68 ± 769.36	1,280.39 ± 678.41	1,263.10 ± 744.45	1,562.53 ± 795.32	0.023	0.423	0.429
Median (IQR)	1,539 (1,082 − 2,032)	1,526 (1,123-1,966)	1,232 (868-1,659)	1,199 (760-1,661)	1,500 (1,066 − 1,987)			
**Physician age**								
Mean ± SD	52.91 ± 12.08	51.88 ± 11.96	51.78 ± 12.22	52.14 ± 11.62	52.54 ± 12.07	0.085	0.093	0.065
Median (IQR)	54 (44–62)	53 (43–61)	53 (42–61)	53 (43–61)	53 (44–62)			
**Physician sex**								
Female	64,747 (40.0%)	23,140 (36.7%)	8,453 (38.2%)	460 (35.4%)	96,800 (39.0%)	0.067	0.037	0.095
Male	97,165 (60.0%)	39,861 (63.3%)	13,697 (61.8%)	839 (64.6%)	151,562 (61.0%)	0.067	0.037	0.095
**Years since graduation**								
<=15	35,249 (21.8%)	16,404 (26.0%)	6,321 (28.5%)	335 (25.8%)	58,309 (23.5%)	0.100	0.156	0.095
16–25	35,513 (21.9%)	13,533 (21.5%)	4,187 (18.9%)	250 (19.2%)	53,483 (21.5%)	0.011	0.075	0.067
26–35	47,752 (29.5%)	18,622 (29.6%)	6,747 (30.5%)	409 (31.5%)	73,530 (29.6%)	0.001	0.021	0.043
36+	43,360 (26.8%)	14,431 (22.9%)	4,892 (22.1%)	305 (23.5%)	62,988 (25.4%)	0.090	0.109	0.076
missing	38 (0.0%)	11 (0.0%)	<=5 (0.0%)	0 (0.0%)	52 (0.0%)	0.004	0.007	0.022
**Practice location**								
Urban	143,528 (88.6%)	13,895 (22.1%)	2,494 (11.3%)	343 (26.4%)	160,260 (64.5%)	1.804	2.444	1.621
Non-major urban	6,269 (3.9%)	41,376 (65.7%)	4,794 (21.6%)	348 (26.8%)	52,787 (21.3%)	1.705	0.553	0.671
Rural	995 (0.6%)	2,783 (4.4%)	12,497 (56.4%)	443 (34.1%)	16,718 (6.7%)	0.245	1.572	0.986
Missing	11,119 (6.9%)	4,947 (7.9%)	2,365 (10.7%)	165 (12.7%)	18,596 (7.5%)	0.038	0.135	0.197
Payment model*								
Blended capitation	61,666 (38.1%)	18,992 (30.1%)	4,675 (21.1%)	217 (16.7%)	85,550 (34.4%)	0.168	0.379	0.494
Comprehensive Care Model	5,100 (3.1%)	2,087 (3.3%)	892 (4.0%)	37 (2.8%)	8,116 (3.3%)	0.009	0.047	0.018
Family Health Group	44,561 (27.5%)	6,701 (10.6%)	1,723 (7.8%)	153 (11.8%)	53,138 (21.4%)	0.440	0.536	0.404
Family Health Team	35,634 (22.0%)	29,384 (46.6%)	11,830 (53.4%)	521 (40.1%)	77,369 (31.2%)	0.537	0.685	0.399
Non-group physician	14,748 (9.1%)	4,234 (6.7%)	2,399 (10.8%)	189 (14.5%)	21,570 (8.7%)	0.089	0.058	0.169
Other	202 (0.1%)	1,603 (2.5%)	631 (2.8%)	182 (14.0%)	2,618 (1.1%)	0.212	0.227	0.563
**Full-time (Full-time equivalent > = 1.00)**	126,652 (78.2%)	48,824 (77.5%)	15,088 (68.1%)	775 (59.7%)	191,339 (77.0%)	0.017	0.230	0.409

* Family physicians in Ontario receive a variety of payment models. We have grouped Family Health Organizations and Family Health Networks into “Blended capitation”. The Comprehensive Care Model and Family Health Group are fee-for-service models enhanced with bonuses and premiums and a small capitation fee. Family Health Teams are an interdisciplinary team model where most family physicians receive blended capitation, but also receive funding for other health professionals. Non-group physicians are not part of a physician group, and mostly receive fee-for-service payment. “Other” payment models are mostly one-off payment models focused on target patient-populations. More information about Ontario’s family physician payment models can be found here: https://www.health.gov.on.ca/en/pro/programs/pcpm/

**Table 3:** Unadjusted rates of follow-up with prescribing family physician within 30 days of first antidepressant prescription (by rurality)

Rurality	n	%
All	84,639	34.08
Urban	58,159	35.92
Non-major urban	19,661	31.21
Rural	6487	29.29

In Table 4, we provide the results of our multivariable regression examining the association between relational continuity and follow-up, stratified by patients living in urban, non-major urban, and rural communities (Supplementary Tables A2 to A5 for full model results). There are negligible differences in the estimates between Models 1 through 3. The fully adjusted rate ratio RRR = 1.005 (95% CI = 1.004, 1.006) can be interpreted as follows: conditional on covariates, for every 1-year increase in relational continuity, the relative risk of receiving follow-up care within 30 days increased on average by 1.005-fold. In the stratified sample, the fully adjusted model yielded the following results: RRR = 1.003 (95% CI = 1.002, 1.005) for patients in urban communities, RRR = 1.009 (95% CI = 1.007, 1.012) in non-major urban communities, and RRR = 1.006 (95% CI = 1.002, 1.011) in rural communities. These findings suggest that residing in more rural communities slightly positively modifies the relationship between relational continuity and follow-up care.

**Table 4:** Estimates of the relationship between relational continuity and prescribing family physician follow-up within 30 days

Stratification	Model 1*			Model 2*			Model 3*		
	Relative Risk Ratio	95% Confidence Limits		Relative Risk Ratio	95% Confidence Limits		Relative Risk Ratio	95% Confidence Limits	
All	1.0050	1.0041	1.0059	1.0062	1.0053	1.0071	1.0051	1.0038	1.0064
Urban	1.0027	1.0016	1.0038	1.0044	1.0033	1.0055	1.0034	1.0019	1.0049
Non-major urban	1.0086	1.0067	1.0106	1.0084	1.0065	1.0104	1.0094	1.0068	1.0120
Rural	1.0114	1.0080	1.0147	1.0115	1.0081	1.0148	1.0064	1.0023	1.0106

* Model 1 incorporated patient-level sociodemographic variables including the interaction of age and sex categories, neighborhood-level material deprivation, and migration status and patient morbidity (CIHI grouper). Model 2 added the number of primary care visits in the prior 24 months and number of unique prescriptions at the dispensing date, and Model 3 added prescribing-physician-level variables (physician sex, physician age, and physician payment model)

We also estimated models (Supplementary Table A6) with a binary exposure where relational continuity was less than five years versus greater than or equal to five years. In the fully adjusted model, patients with relational continuity ≥ 5 years were 1.067 (95% CI = 1.046, 1.086) times more likely to receive follow-up care.

A secondary outcome measured follow-up with *any* FP within 30-days (Supplementary Table A7). For this outcome, the relationship was reversed RRR = 0.995 (95% CI = 0.995, 0.996), but the magnitude of the effects for both exposures remained small. Locally estimated scatterplot smoothing (LOESS) plots (Supplementary Figure A1) show that the relationship between the relational continuity and follow-up is relatively flat for 30-day follow-up with the *prescribing* FP or *any* FP.

## Discussion

In this study, we found a small positive relationship between our measure of relational continuity and follow-up care with the prescribing FP after being dispensed a first antidepressant prescription. This finding is consistent with our initial hypothesis. However, it is important to note that the effect size was small (< 1% relative increase in the likelihood of 30-day follow-up per year of relational continuity). We also found that the relationship between our measure of relational continuity was moderated by the patients’ location of dwelling. Compared to urban communities, the effect size was larger for older adults residing in non-major urban and rural communities.

We could not find many studies with comparable results. Houle et al. [8] found that follow-up care was quite regular in their population of adults in the Montreal area who received a depression diagnosis; 90% of people newly diagnosed with depression consulted with a FP or psychiatrist within 30 days. By comparison, our data indicate that only 34% of older adults had a follow-up visit with the prescribing FP within 30 days of a first prescription, and 56% had a visit with any physician. Although our study focused on follow-up with the prescribing FP among older adults — not adults newly diagnosed with depression — our results are generalizable to all older adults starting antidepressants for any indication. Houle et al. [8] also found that people 65 years of age and older were less likely to receive follow-up care and to have high levels of continuity with their regular provider, but they did not assess the relationship between continuity and follow-up. Massamba et al. [25] used survey data to assess follow-up care after a first antidepressant prescription in older adults in Quebec. They did not observe any differences in the Usual Provider of Care Index – a measure of continuity of care that calculates the proportion of visits performed by the provider that the patient visited most frequently – between older adults who received follow-up care and those who did not. However, the authors used a self-report dataset, had a smaller sample (*n* = 263), and did not assess a measure of relational continuity. Thus, more research on the relationship between continuity and follow-up for depression care is needed to validate our results.

Our study has several limitations. First, our exposure variable captures only one part of relational continuity: the duration of the relationship between the patient and provider. The exposure does not capture the number of contacts during the duration of the relationship. Furthermore, there is no guarantee that a long or intensive relationship will translate to a trusting partnership between a patient and physician [26]. While we do find a relationship between continuity and follow-up care, this signal could be diluted by our inability to measure the quality of the patient-provider relationship in the administrative data. Second, our data do not examine actual use of antidepressants; rather, we can only observe dispensing of prescription medications. Third, given data limitations, we were only able to observe up to 20 years of relational continuity. However, we think this is likely sufficient time to identify a long-term relationship between provider and patient. Fourth, because our study relies on observational data, we are likely missing key explanatory variables (e.g., education and individual-level socioeconomic status) that may be important confounders of the relationship between continuity and follow-up care. Thus, our results should not be interpreted causally. Fifth, the results were reversed when analyzing 30-day follow-up with any FP which is counter-intuitive but is likely explained by the flat relationship between years of relational continuity and likelihood of 30-day follow-up.

Our study has several strengths. We accessed health administrative health data that contain all cases of publicly insured antidepressant medication dispensed to older adults in Ontario. This is a large and representative sample of the population of interest. We were also able to rely on datasets that stretch back in time for decades so that we could construct our exposure of interest. We have operationalized longitudinal/relational continuity, a relatively under-studied component of continuity of care in family practice and identified a need for more nuanced measures of this metric. Furthermore, this study addresses a largely unexplored and policy-relevant question.

Our findings suggest that relational continuity of care is weakly associated with the likelihood of follow-up by the prescribing FP in older adults prescribed antidepressants. There are many possible explanations for the weak association that require exploration in further research. As noted above, the relationship may be diluted by the limitations in assessing the quality of the patient-provider relationship in the administrative data, or by the number of previous contacts the patient had with the prescribing physician. Moreover, the relationship may be moderated by factors such as providers’ practice style, their familiarity with antidepressant care, or patients’ perceived necessity for follow-up, particularly for those with mild symptoms. Notably, Prins et al. [27] found that patients’ perceived need for care predicted receiving guideline-concordant care for depression and anxiety. Our results also suggest that the relationship between continuity and follow-up care was more important in rural communities, where access to continuous primary care and specialized mental health services is more limited [28]. This provides support for the recruitment and retention of primary care providers in rural communities. Future research could focus on younger age groups and those without prescription drug insurance. Additionally, it would be worthwhile to use administrative data to investigate whether relational continuity of care is associated with other measures of follow-up care for individuals diagnosed with depression and anxiety disorders. Qualitative research, such as interviews with patients and prescribers, could also investigate the importance of relational continuity on trust with a prescribing FP and the experiences of receiving or delivering follow-up after a new antidepressant prescription.

### Electronic supplementary material

Below is the link to the electronic supplementary material.

Supplementary Material 1

Supplementary Material 2

## Data Availability

The dataset from this study is held securely in coded form at ICES. While legal data sharing agreements between ICES and data providers (e.g., healthcare organizations and government) prohibit ICES from making the dataset publicly available, access may be granted to those who meet pre-specified criteria for confidential access, available at www.ices.on.ca/DAS (email: das@ices.on.ca). The full dataset creation plan and underlying analytic code are available from the authors upon request, understanding that the computer programs may rely upon coding templates or macros that are unique to ICES and are therefore either inaccessible or may require modification.

## Abbreviations

FP: Family Physician
SSRI: Selective serotonin reuptake inhibitor
ODBP: Ontario Drug Benefit Program
CIHI: Canadian Institute for Health Information
RIO: Rurality Index of Ontario
