# Supplementary material for: The relationship between relational continuity and family physician follow-up after an antidepressant prescription in older adults: a retrospective cohort study
Source: BMC Prim Care. 2024 Apr 22;25:125. doi: 10.1186/s12875-024-02361-0 (PMC11034035; doi:10.1186/s12875-024-02361-0)

### Supplementary Materials

| **Table A1 - Unadjusted rates of follow-up with a physician within 30 days of first antidepressant prescription (by physician type)** | | |
| --- | --- | --- |
| Rurality | n | % |
| Any physician | 140050 | 56.39 |
| Any primary care physician | 107582 | 43.32 |
| Any psychiatrist | 2784 | 1.12 |
| Prescribing primary care physician | 84641 | 34.08 |

| **Table A2 - Full multivariable regression results for all older adults** | | | | |
| --- | --- | --- | --- | --- |
| Variable |  | Relative Risk Ratio | 95% Confidence Limits | |
| Intercept |  | 0.0052 | 0.0048 | 0.0057 |
| Relational continuity | | 1.0051 | 1.0038 | 1.0064 |
| Age-Sex Categories | Female 66-69 (reference) |  |  |  |
|  | Female 70-74 | 1.1038 | 1.0830 | 1.1250 |
|  | Female 75-79 | 1.1369 | 1.1130 | 1.1613 |
|  | Female 80-84 | 1.1705 | 1.1434 | 1.1981 |
|  | Female 85+ | 1.1468 | 1.1178 | 1.1767 |
|  | Male 66-69 | 1.0188 | 0.9951 | 1.0429 |
|  | Male 70-74 | 1.1172 | 1.0904 | 1.1447 |
|  | Male 75-79 | 1.1777 | 1.1477 | 1.2085 |
|  | Male 80-84 | 1.1898 | 1.1560 | 1.2246 |
|  | Male 85+ | 1.2107 | 1.1732 | 1.2493 |
|  | No condition (reference) | - | - | - |
|  | Major-palliative | 1.3268 | 1.2184 | 1.4448 |
|  | Moderate | 1.3752 | 1.2636 | 1.4965 |
|  | Minor | 1.3328 | 1.2268 | 1.4479 |
|  | Non-User | 1.4851 | 1.3114 | 1.6820 |
| Neighborhood material deprivation | 1 (reference) | - | - | - |
|  | 2 | 0.9991 | 0.9822 | 1.0164 |
|  | 3 | 0.9644 | 0.9470 | 0.9823 |
|  | 4 | 0.9852 | 0.9675 | 1.0031 |
|  | 5-most deprived | 0.9479 | 0.9301 | 0.9661 |
|  | 6 - missing | 0.9417 | 0.8754 | 1.0130 |
| Recent migrant |  | 1.0195 | 0.9835 | 1.0569 |
| No. of unique drugs | 0-5 (reference) |  |  |  |
|  | 6-9 | 1.0751 | 1.0527 | 1.0979 |
|  | 10-19 | 1.1928 | 1.1633 | 1.2230 |
|  | 20+ | 1.2830 | 1.2304 | 1.3378 |
| No. of FP visits in last 24 months |  | 1.0128 | 1.0101 | 1.0154 |
| Male physician |  | 1.0220 | 1.0050 | 1.0394 |
| Physician age |  | 0.9995 | 0.9986 | 1.0003 |
| Payment model | Blended capitation (reference) | - | - | - |
|  | Comprehensive Care Model | 1.2456 | 1.1987 | 1.2943 |
|  | Family Health Group | 1.2151 | 1.1870 | 1.2438 |
|  | Family Health Team | 0.9509 | 0.9306 | 0.9717 |
|  | Non-Group Physician | 0.9480 | 0.9127 | 0.9847 |
|  | Other | 0.9216 | 0.8412 | 1.0096 |

| **Table A3 - Full multivariable regression results for older adults in urban communities** | | | | |
| --- | --- | --- | --- | --- |
| Variable |  | Relative Risk Ratio | 95% Confidence Limits | |
| Intercept |  | 0.0052 | 0.0046 | 0.0059 |
| Relational continuity | | 1.0034 | 1.0019 | 1.0049 |
| Age-sex Categories | Female 66-69 (reference) | - | - | - |
|  | Female 70-74 | 1.0972 | 1.0732 | 1.1220 |
|  | Female 75-79 | 1.1278 | 1.1002 | 1.1562 |
|  | Female 80-84 | 1.1529 | 1.1225 | 1.1841 |
|  | Female 85+ | 1.1221 | 1.0890 | 1.1562 |
|  | Male 66-69 | 1.0164 | 0.9883 | 1.0454 |
|  | Male 70-74 | 1.1119 | 1.0804 | 1.1443 |
|  | Male 75-79 | 1.1716 | 1.1363 | 1.2080 |
|  | Male 80-84 | 1.1560 | 1.1167 | 1.1967 |
|  | Male 85+ | 1.1983 | 1.1558 | 1.2422 |
|  | No condition (reference) | - | - | - |
|  | Major-palliative | 1.3893 | 1.2418 | 1.5543 |
|  | Moderate | 1.4446 | 1.2919 | 1.6151 |
|  | Minor | 1.4165 | 1.2686 | 1.5819 |
|  | Non-User | 1.6489 | 1.4145 | 1.9219 |
| Neighborhood material deprivation | 1 (reference) | - | - | - |
|  | 2 | 1.0019 | 0.9817 | 1.0227 |
|  | 3 | 0.9739 | 0.9535 | 0.9948 |
|  | 4 | 1.0000 | 0.9792 | 1.0211 |
|  | 5-most deprived | 0.9548 | 0.9341 | 0.9760 |
|  | 6 - missing | 1.1219 | 1.0076 | 1.2491 |
| Recent migrant |  | 1.0119 | 0.9740 | 1.0512 |
| No. of unique drugs | 0-5 (reference) | - | - | - |
|  | 6-9 | 1.0699 | 1.0436 | 1.0970 |
|  | 10-19 | 1.1790 | 1.1461 | 1.2130 |
|  | 20+ | 1.2484 | 1.1943 | 1.3050 |
| No. of FP visits in last 24 months |  | 1.0121 | 1.0094 | 1.0147 |
| Male physician |  | 1.0287 | 1.0094 | 1.0483 |
| Physician age |  | 0.9997 | 0.9987 | 1.0006 |
| Payment model | Blended capitation (reference) | - | - | - |
|  | Comprehensive Care Model | 1.2191 | 1.1658 | 1.2749 |
|  | Family Health Group | 1.2062 | 1.1769 | 1.2361 |
|  | Family Health Team | 0.9251 | 0.9005 | 0.9504 |
|  | Non-Group Physician | 0.9727 | 0.9344 | 1.0126 |
|  | Other | 0.9834 | 0.7721 | 1.2526 |

| **Table A4 - Full multivariable regression results for older adults in non-major urban communities** | | | | |
| --- | --- | --- | --- | --- |
| Variable |  | Relative Risk Ratio | 95% Confidence Limits | |
| Intercept |  | 0.0054 | 0.0046 | 0.0065 |
| Relational continuity | | 1.0094 | 1.0068 | 1.0120 |
| Age-sex categories | Female 66-69 (reference) | - | - | - |
|  | Female 70-74 | 1.1010 | 1.0593 | 1.1443 |
|  | Female 75-79 | 1.1396 | 1.0936 | 1.1876 |
|  | Female 80-84 | 1.1860 | 1.1304 | 1.2443 |
|  | Female 85+ | 1.1823 | 1.1258 | 1.2417 |
|  | Male 66-69 | 1.0293 | 0.9869 | 1.0735 |
|  | Male 70-74 | 1.1187 | 1.0669 | 1.1732 |
|  | Male 75-79 | 1.1682 | 1.1089 | 1.2306 |
|  | Male 80-84 | 1.2404 | 1.1733 | 1.3114 |
|  | Male 85+ | 1.1860 | 1.1113 | 1.2658 |
|  | No condition (reference) | - | - | - |
|  | Major-palliative | 1.1613 | 0.9982 | 1.3509 |
|  | Moderate | 1.2226 | 1.0535 | 1.4191 |
|  | Minor | 1.1870 | 1.0230 | 1.3774 |
|  | Non-User | 1.1367 | 0.8564 | 1.5086 |
| Neighborhood material deprivation | 1 (reference) | - | - | - |
|  | 2 | 1.0041 | 0.9683 | 1.0411 |
|  | 3 | 0.9761 | 0.9395 | 1.0140 |
|  | 4 | 0.9879 | 0.9486 | 1.0287 |
|  | 5-most deprived | 0.9452 | 0.9067 | 0.9853 |
|  | 6 - missing | 0.8828 | 0.7586 | 1.0273 |
| Recent migrant |  | 0.8877 | 0.7804 | 1.0096 |
| No. of unique drugs | 0-5 (reference) | - | - | - |
|  | 6-9 | 1.0924 | 1.0449 | 1.1421 |
|  | 10-19 | 1.2131 | 1.1602 | 1.2686 |
|  | 20+ | 1.3117 | 1.2380 | 1.3897 |
| No. of FP visits in last 24 months |  | 1.0184 | 1.0153 | 1.0215 |
| Male physician |  | 1.0203 | 0.9845 | 1.0574 |
| Physician age |  | 0.9970 | 0.9951 | 0.9988 |
| Payment model | Blended capitation (reference) | - | - | - |
|  | Comprehensive Care Model | 1.2876 | 1.2018 | 1.3796 |
|  | Family Health Group | 1.2246 | 1.1642 | 1.2880 |
|  | Family Health Team | 1.0469 | 1.0055 | 1.0898 |
|  | Non-Group Physician | 0.9109 | 0.8433 | 0.9839 |
|  | Other | 0.9283 | 0.8341 | 1.0331 |

| **Table A5 - Full multivariable regression results for older adults in rural communities** | | | | |
| --- | --- | --- | --- | --- |
| Variable |  | Relative Risk Ratio | 95% Confidence Limits | |
| Intercept |  | 0.0047 | 0.0036 | 0.0061 |
| Relational continuity | | 1.0064 | 1.0023 | 1.0106 |
| Age-sex categories | Female 66-69 (reference) | - | - | - |
|  | Female 70-74 | 1.1043 | 1.0294 | 1.1847 |
|  | Female 75-79 | 1.1426 | 1.0577 | 1.2342 |
|  | Female 80-84 | 1.1574 | 1.0626 | 1.2609 |
|  | Female 85+ | 1.1216 | 1.0234 | 1.2295 |
|  | Male 66-69 | 1.0280 | 0.9515 | 1.1106 |
|  | Male 70-74 | 1.1499 | 1.0560 | 1.2522 |
|  | Male 75-79 | 1.2088 | 1.1079 | 1.3186 |
|  | Male 80-84 | 1.2556 | 1.1394 | 1.3836 |
|  | Male 85+ | 1.2023 | 1.0521 | 1.3737 |
|  | No condition (reference) | - | - | - |
|  | Major-palliative | 1.1882 | 0.9542 | 1.4795 |
|  | Moderate | 1.1590 | 0.9363 | 1.4348 |
|  | Minor | 1.0790 | 0.8732 | 1.3331 |
|  | Non-User | 1.2281 | 0.8255 | 1.8272 |
| Neighborhood material deprivation | 1 (reference) | - | - | - |
|  | 2 | 1.0028 | 0.9258 | 1.0862 |
|  | 3 | 0.9670 | 0.8947 | 1.0451 |
|  | 4 | 0.9208 | 0.8504 | 0.9969 |
|  | 5-most deprived | 0.9353 | 0.8573 | 1.0205 |
|  | 6 - missing | 0.9508 | 0.7981 | 1.1329 |
| Recent migrant |  | 1.1797 | 0.9775 | 1.4239 |
| No. of unique drugs | 0-5 (reference) | - | - | - |
|  | 6-9 | 1.0215 | 0.9502 | 1.0981 |
|  | 10-19 | 1.1058 | 1.0323 | 1.1847 |
|  | 20+ | 1.1536 | 1.0488 | 1.2688 |
| No. of FP visits in last 24 months |  | 1.0219 | 1.0176 | 1.0262 |
| Male physician |  | 1.0133 | 0.9531 | 1.0773 |
| Physician age |  | 1.0027 | 0.9998 | 1.0055 |
| Payment model | Blended capitation (reference) | - | - | - |
|  | Comprehensive Care Model | 1.1947 | 1.0666 | 1.3383 |
|  | Family Health Group | 1.0814 | 0.9829 | 1.1897 |
|  | Family Health Team | 0.9533 | 0.8957 | 1.0148 |
|  | Non-Group Physician | 0.8542 | 0.7435 | 0.9814 |
|  | Other | 1.1547 | 0.9871 | 1.3505 |

| **Table A6 - Full multivariable regression results for all older adults (binary exposure relational continuity <5 years, >= 5 years)** | | | | |
| --- | --- | --- | --- | --- |
| Variable |  | Relative Risk Ratio | 95% Confidence Limits | |
| Intercept |  | 0.0051 | 0.0047 | 0.0056 |
| Relational continuity | | 1.0658 | 1.0455 | 1.0864 |
| Age-sex categories | Female 66-69 (reference) | - | - | - |
|  | Female 70-74 | 1.1034 | 1.0826 | 1.1246 |
|  | Female 75-79 | 1.1363 | 1.1125 | 1.1607 |
|  | Female 80-84 | 1.1702 | 1.1433 | 1.1978 |
|  | Female 85+ | 1.1459 | 1.1171 | 1.1756 |
|  | Male 66-69 | 1.0179 | 0.9943 | 1.0419 |
|  | Male 70-74 | 1.1156 | 1.0889 | 1.1431 |
|  | Male 75-79 | 1.1763 | 1.1464 | 1.2070 |
|  | Male 80-84 | 1.1891 | 1.1552 | 1.2238 |
|  | Male 85+ | 1.2099 | 1.1725 | 1.2483 |
|  | No condition (reference) | - | - | - |
|  | Major-palliative | 1.3288 | 1.2202 | 1.4472 |
|  | Moderate | 1.3778 | 1.2660 | 1.4995 |
|  | Minor | 1.3348 | 1.2286 | 1.4501 |
|  | Non-User | 1.4811 | 1.3077 | 1.6775 |
| Neighborhood material deprivation | 1 (reference) | - | - | - |
|  | 2 | 0.9998 | 0.9828 | 1.0170 |
|  | 3 | 0.9650 | 0.9475 | 0.9828 |
|  | 4 | 0.9856 | 0.9680 | 1.0035 |
|  | 5-most deprived | 0.9483 | 0.9303 | 0.9665 |
|  | 6 - missing | 0.9415 | 0.8751 | 1.0128 |
| Recent migrant |  | 1.0075 | 0.9723 | 1.0441 |
|  | Missing | 1.2295 | 0.8670 | 1.7437 |
| No. of unique drugs | 0-5 (reference) | - | - | - |
|  | 6-9 | 1.0749 | 1.0525 | 1.0977 |
|  | 10-19 | 1.1924 | 1.1630 | 1.2227 |
|  | 20+ | 1.2818 | 1.2292 | 1.3367 |
| No. of FP visits in last 24 months |  | 1.0127 | 1.0101 | 1.0154 |
| Male physician |  | 1.0235 | 1.0064 | 1.0407 |
| Physician age |  | 1.0001 | 0.9993 | 1.0009 |
| Payment model | Blended capitation (reference) | - | - | - |
|  | Comprehensive Care Model | 1.2422 | 1.1957 | 1.2907 |
|  | Family Health Group | 1.2132 | 1.1853 | 1.2418 |
|  | Family Health Team | 0.9490 | 0.9289 | 0.9698 |
|  | Non-Group Physician | 0.9472 | 0.9120 | 0.9838 |
|  | Other | 0.9150 | 0.8354 | 1.0024 |

| **Table A7 - Estimates of the relationship between interpersonal continuity and any family physician follow-up within 30 days** | | | | | | | | | |
| --- | --- | --- | --- | --- | --- | --- | --- | --- | --- |
|  | Model 1 |  |  | Model 2 |  |  | Model 3 |  |  |
| **Stratification** | Relative Risk Ratio | 95% Confidence Limits | | Relative Risk Ratio | 95% Confidence Limits | | Relative Risk Ratio | 95% Confidence Limits | |
| All | 0.9953 | 0.9945 | 0.9961 | 0.9966 | 0.9958 | 0.9975 | 0.9960 | 0.9951 | 0.9969 |

Figure A1 - LOESS curves showing the relationship between relational continuity and the average rate of 30-day physician follow-up.


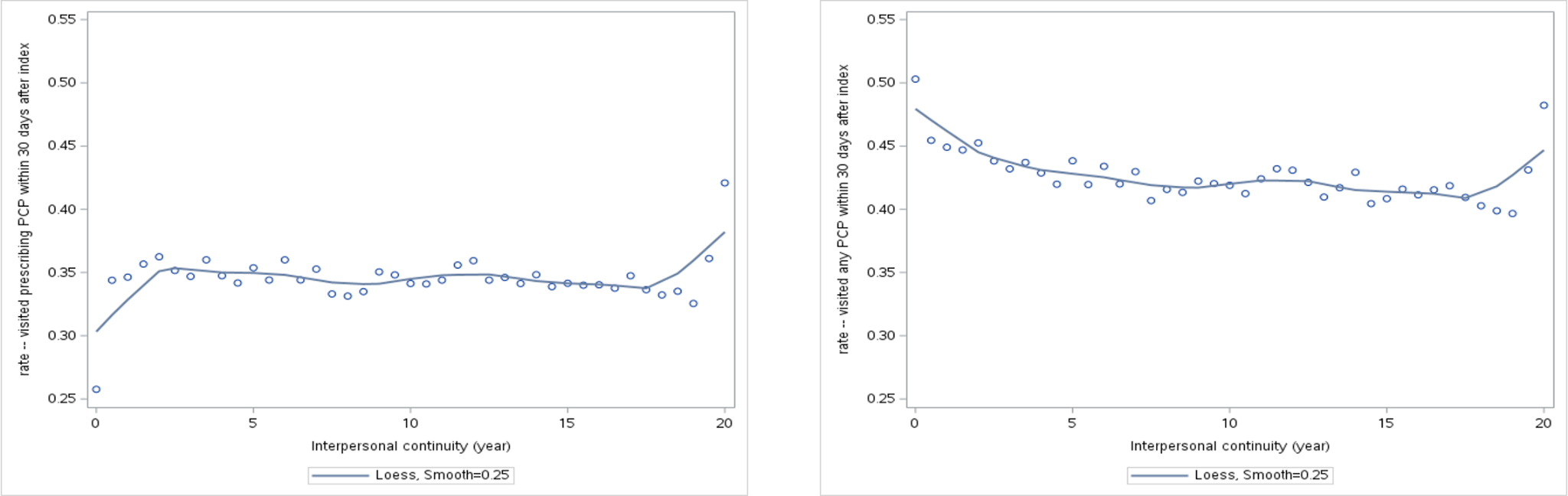

Supplement: Supplementary file 2 — Supplementary Material 2 [file 12875_2024_2361_MOESM2_ESM.docx]
